# ‘Humanizing’ healthcare environments: architecture, art and design in modern hospitals

**DOI:** 10.1080/24735132.2018.1436304

**Published:** 2018-02-15

**Authors:** Victoria Bates

**Affiliations:** Department of History, University of Bristol, Bristol, United Kingdom

**Keywords:** Humanistic design, hospital architecture, interior design, arts and health, nature, homeliness

## Abstract

In recent decades, hospital design literature has paid increasing attention to an apparent need to ‘humanize’ hospital environments. Despite the prevalence of this design goal, the concept of ‘humanizing’ a space has rarely been defined or interrogated in depth. This article focuses on the meaning of humanization, as a necessary step towards understanding its implementation in practice. It explores the recent history of humanistic design as a goal in healthcare contexts, focusing on the UK in the late twentieth century. It shows that many features of humanistic design were not revolutionary, but that they were thought to serve a new purpose in counterbalancing high-technology, scientific and institutional medical practice. The humanistic hospital, as an ideal, operated as a symbol for wider social concerns about the loss – or decentring – of patients in modern medical practice. Overall, this article indicates a need to interrogate further the language of ‘humanization’ and its history. The term is not value free; it carries with it assumptions about the dehumanization of modern medicine, and has often been built on implicit binaries between the human and the technological.

## Introduction

In the early 1980s, an article in the *British Medical Journal* (*BMJ*) made the following passing comment: ‘of course we must humanise hospitals’ (Bardon 1981). Its casual use of ‘of course’ is highly revealing, implying that this comment was made in a context of general acceptance that hospital care needed reform. Calls to (re)humanize hospitals in the UK had indeed increased over the post-war period, and were part of wider international trends. In 1958, the French Ministry of Health had produced a circular on ‘humanisation des hôpitaux’, which articulated the need for humanistic hospital care under a system of universal health care (Nardin 2009).^1.^ Nor were these concerns a product only of welfare states. In 1981, architect James Falick wrote an article for *Hospitals* journal under the heading ‘Humanistic Design Sells your Hospital’, arguing for the value of paying attention to hospital aesthetics as well as function in the more consumer-driven United States. By the end of the century projects seeking to humanize hospitals were evident across the globe, from Japan to Brazil (Lewis 1983; Misago et al. 1999; Santos and Siebert 2001). Some of these initiatives focused on daily life in hospitals or on specific contexts of apparent ‘dehumanization’, most commonly maternity care. Many others advocated the humanization of hospitals in broad terms, which included reference to hospital architecture, layout and interior design.

References to ‘humanizing’ hospitals were made in a range of social and political contexts across the world, a fact that raises some questions about the meaning of the term. Humanistic design undoubtedly had very different implications when its end goal was to support universal hospital care in France in the 1950s, rather than to ‘sell’ a US hospital in the 1980s, but few commentators ever drew out such differences. The term was widely cited across different decades, countries, healthcare systems and types of patient in the late twentieth century, but often uncritically. Despite the increasing influence of humanization as a design goal, the concept remains under examined. This article seeks to unpick some of the different ways in which the term has been used in this period, in order to understand better its rhetorical power and continued influence over hospital design. It focuses on ideas about humanistic design in UK medical and design literature under the new National Health Service (NHS) in the late twentieth century, a context that drew on design theory both from Europe and the United States. The article also makes comparisons with these geographical contexts, where relevant, to highlight similarities and differences in the meanings of humanistic design.

The discussion focuses on the spread of humanization as an idea, rather than its implementation. The multiple meanings and ideological underpinnings of ‘humanistic design’ are worthy of detailed attention in their own right. Part of the value of understanding this language is, of course, that it will enable a better understanding of trends in ‘humanistic’ healthcare design in practice. However, it is important also to remember that the growing influence of an *idea* and *practice* are not synonymous. Indeed, for practical and economic reasons, there were a number of limits on the extent to which ‘humanistic’ design principles could be implemented in practice, but this does not undermine their ideological significance. In many ways, the principles underpinning humanistic design were nothing new. It remains significant, though, that late twentieth-century medical and architectural literature presented some of these fairly traditional design features in the new language of ‘(re)humanization’. This terminology is highly revealing in its implicit opposition to the ‘dehumanized’ modern hospital, which operated as a material representation of modern medical practice (itself also a construct in many ways). Such fears about the loss of the ‘human’ aspects of medicine with the rise of technologies also have a long history in the UK, but grew exponentially in the wake of the Second World War.

The article begins with a brief history of the human as a design goal in healthcare environments, and an overview of its different forms. Its second section considers three different ways in which the human has been defined by its opposition to features of the modern, dehumanized hospital and, implicitly, to aspects of modern medical practice. These different forms of ‘humanization’ were bound together by a shared goal of person-centred, rather than science- or technology-centred, medicine. The ‘human’ aspects of design have increasingly sought to address the needs of staff and visitors, but patients have been the primary focus of concerns about ‘dehumanization’ and its effects (see Bromley 2012; Gesler et al. 2004). The ‘human’ operated as an umbrella term in opposition to constructions of ‘dehumanized’ medical care, with its apparent loss of patient agency, individualism and holism. With the continued prevalence of the language of humanization in healthcare design today, the political and social contexts in which these ideas were initially produced must be acknowledged.

## The human as design goal: a brief history

In 2005, the South Tees Acute Hospitals NHS Trust published a study of the ‘art and science of creating environments that prevent illness, speed healing and promote well-being’ (Macnaughton et al. 2005). *Designing for Health*, this publication argued, necessitated ‘humanis[ing] the “inhospitable” hospital’ in a range of ways, including providing a sense of control, external views, positive acoustics, natural light, pleasant fragrances, bodily comfort, varied colour and private space. The length of this list indicates the wide range of different design features considered to humanize a space, and the way in which the term is often used as an umbrella to include anything that makes a hospital less ‘inhospitable’. This section seeks briefly to outline how and why so many features of hospital design came to be clustered under the heading of humanistic design in the late twentieth century. It also considers why such design was thought particularly necessary at this time.

Historicizing the language of ‘humanistic’ medicine is a significant task, due to its overlaps – and sometimes interchangeable use – with terms such as ‘humanitarian’, ‘humanism’, ‘humanity’, among others (Penman and Adams 1982; Kopelman 1999). It is not possible to do justice to these linguistic complexities here. However, it is worthy of note that the very language of humanistic medicine carried with it a much longer history of ideas about good medical care and the relationships between healthcare practitioners, patients and communities. Its root alone, ‘human’, carries with it the weight of big questions about ‘what makes us human?’ that connect to themes such as – among others – human rights, identities, emotions, creativity, sensibility and culture (Bourke 2011). The concepts of humanization and dehumanization in hospital design often rested, albeit almost always implicitly, upon culturally specific assumptions about what the human was and – in particular – what it was *not*.

This article's focus on the late twentieth century is not to claim that humanistic design was an entirely new phenomenon in this period. Ideas about the importance of holistic models of health care, including healthcare environments, of the value of spaces for reflection when unwell, of cheerful spaces and distraction for the ill, and of the healing powers of nature can be traced back for centuries (Hickman 2009). *The Lancet* commented in 1866 on the ‘humanising influence’ of ‘neatness and beauty of arrangement in the wards’ of one typhus hospital (Anon. 1866). A number of the features later identified as ‘humanistic’ ideals were also evident in famous examples of Victorian design, such as Florence Nightingale's emphasis on visual stimulation, nature and colour in wards. They were also found in some outstanding early twentieth-century modernist hospitals, despite the tendency later to align modernism with ‘dehumanization’, such as Alvar Aalto's famous Paimio Sanitorium in Finland and Berthold Lubetkin's Finsbury Health Centre in London.

It is possible to keep going back further in history, across time and place, identifying such examples of ‘humanistic’ design. To claim that humanization was a particularly important idea in late twentieth-century design is, therefore, not to claim that it marked a complete break from earlier paradigms of health and design. These longer histories are key to understanding why the ‘humanistic’ value of certain design features – such as arts, gardens and homeliness – were rarely questioned. ‘Humanization’ also, though, represented something new. These design features took on a specific meaning in relation to the language of ‘dehumanization’ and a perceived need to (re)human*ize* hospitals, which indicated a new sense of loss. Humanistic design was constructed in opposition to particular features of modern medicine, particularly technologies and practices that diminished the patient's voice or individualism. Such concerns about the loss of the individual may have reflected some of the wider – albeit highly complex and much debated – tensions surrounding modernity itself and its impact on the individual; modernity, capitalism, industrialism and globalization simultaneously encouraged and threatened individualism.^2.^ It is also important to acknowledge that this model of the ‘human’ is culturally specific, with its focus on the individual and self-expression.

The language of humanization seemed to grow in a wide range of contexts at a similar time, often as part of wider critiques of Western health care and big institutions. These trends related in part to social shifts that promoted social equality and sought to provide voices for the marginalized (including patients) in the 1960s and 1970s. Some work in this area came from philosopher and priest Ivan Illich, for example, who is well known for critiquing the medicalization of society and medical iatrogenesis in *Medical Nemesis* (1975); Illich travelled widely and his ideas gained significant attention in Europe and the United States. Perhaps less well known is that Illich published similar ideas in an edited collection entitled *Humanizing Hospital Care* (Turner and Mapa 1979), showing how interwoven these wider critiques of medicine were with the hospital humanization agenda. Although this collection focused on human relationships in hospitals, rather than design, these two issues have never really been separable. Many of these broad, international contemporary critiques of modern medicine came from outside of the profession. In the 1960s and 1970s, they raised concerns about the growing power of medical knowledge, the rise of technology and the consequent shift of patient from subject to object, and the shift towards large impersonal large institutions. Many of these works focused on psychiatric institutions (for example, Goffman 1961), but others made broader critiques of medical knowledge about the body and of clinical spaces (for example, Foucault 1973 [1963]). As a high technology space, the built environment of the hospital came physically to represent many of these wider critiques.

Mechanization and technology were particularly prominent themes of literature on medical ‘dehumanization’, and on wider social dehumanization. Concerns about the negative impact of technology on human relationships and on health in general had a longer history, famously articulated through modern disease categories such as the famous late-Victorian ‘railway spine’ and First World War ‘shell shock’. In the hospital context, the health benefits of technology were rarely in doubt, but the implications of high-technology environments for experience and care – as opposed to cure – became increasingly central themes of discussions about hospital operations and design. According to those who made such critiques, the (increasingly voiceless) patient's body was reconceptualized in modernity as a system, with its faulty parts to be identified and repaired in the hospital (now a ‘machine for healing’). As one editorial commented in the *American Journal of Public Health* in 1978, neatly summarizing the prevailing intellectual mood, ‘for the past century, the more rapid the advance has been in technology, the less emphasis there has been on the caring and human aspects of medical practice’ (Korsch 1978). These kinds of comments relied on the construction of binaries, between care/humanity and cure/technology. Such ideas were undoubtedly problematic in many ways, and some contemporaries recognized them as such. In a sociology-driven article for *Medical Care* journal, Howard et al. (1977) argued that ‘technologies of the health-care industry can be humanizing or dehumanizing depending on how and for whom they are applied’, but that they still had a negative reputation. ‘In light of its humanizing potential’, they asked ‘why is technology so often considered a crucial source of dehumanization?’ In seeking to challenge this idea, though, they acknowledged its power and prevalence.

Humanization was also emerging as an agenda in wider medical practice, including education, and in other architectural contexts such as workplace design. One Canadian commentator noted the link between hospital and wider design trends, when it wrote that ‘internationally renowned architect Eberhard Zeidler … saw increasingly complex hospital technology and scientific approach threatening to turn [the] vulnerable human into a faceless case. It is not mere coincidence that, in the wider world, the word in office design today is “humanize”’ (Kimball 1984). Concerns about the human aspects of design and health were thus part of a wider culture shift – with circulation of ideas between the United States, Canada and Europe – and were not limited to the medical sphere.

In the UK, such trends were connected to the rise of the welfare state and its more egalitarian model of public services provision, in which the human was collective as well as individual. Various practical considerations also necessitated the rethink of hospital design under the new NHS, shaping the specific meaning of ‘humanization’ in the UK. The NHS had inherited very diverse hospitals, with a wide range of design features and daily practices, and needed to make these serve the purposes of the entire population for the first time. In moving towards understanding and standardizing these hospitals, the Ministry of Health and the independent King's Fund health charity commissioned several reports to understand how hospitals operated and their impact on patients. These reports, which gathered extensive feedback from staff and patients, indicate that pressure for change was not always driven by external forces. As David Armstrong (1998) notes, this ‘series of reports and publications during the 1950s and early 1960s proposed measures to humanise the hospital’. Armstrong argues that there was no specific political or economic agenda to these studies, but that they resulted in ‘a wide-ranging technical critique of the hospital’. Many of these proposed measures related to daily routines, but many others related specifically to hospital aesthetics. Humanizing medical practice and care in hospitals was inseparable from the environment in which that care took place.

Overall, the concept of humanistic hospital design was inextricably interwoven with ideas about good medical care. In some ways it emerged as part of large international, cultural shifts, and in others was shaped by specific national and local contexts including patients’ own voices. There were some common features to these calls for ‘humanistic’ hospital care and design. One was a tendency for voices from outsides the medical profession to be those calling for change, even though many medical practitioners were also receptive to these ideas. The second was a shared emphasis on putting the patient back at the centre of hospital design. The perceived need to re-humanize hospital spaces was grounded in a sense of loss, in terms of patients’ apparent increasing voicelessness and lack of visibility as both individuals and communities, in modern medical care. While undoubtedly a simplistic construction of modern hospital medicine, and not one that was universally accepted, it had power. This context is crucial for understanding the very wide range of design factors labelled as humanistic in the late twentieth century, which at first seem to have very little in common beyond a general goal of aesthetic improvement. In practice, though, they were bound together in opposition to the ‘machine for healing’: a symbol of modern healthcare practices.

## Scale and the soul: the human in hospital design

The human aspects of hospital design stood together in opposition to a particular construction of modern medical practice, but they took multiple forms. This section will explore three of the different aspects of humanistic design principles, considering how they operated in opposition to three different types of modern medical practice in the UK. First, it considers the human as not-institutional; this type of humanistic space took form through the idea of human scale and modelling hospital spaces on the village or the home. Second, it considers the human as not-technology; this humanization operated through hiding technologies and prioritizing natural sensescapes in the hospital. Finally, the article considers the human as not-biomedical; this form of humanistic design necessitated environments that addressed patients’ emotional and holistic needs. These three forms of ‘humanistic’ design undoubtedly overlapped, but were also distinct in some ways: the term operated as an umbrella, embracing all design features that opposed modern ‘dehumanized’ medical practice. This implicit opposition to ‘dehumanized’ medicine gave ‘humanization’ its rhetorical and ideological power in late twentieth-century design literature.

Reducing the scale of buildings was one important goal of humanistic architecture in the UK. It was in line with European intellectual trends, many of which situated the human in opposition to the modern and contemporary rise of large medical (and other) institutions (for example, Foucault 1965 [1961]). Concerns about scale also reflected the problem of a growing number of hospital beds in existing buildings. During the initial post-war hospital shortage, medical journal *The Lancet* reported that a medical officer from the Ministry of Health argued for the number of beds in hospitals to be limited on ‘humanistic grounds’ (Anon. 1947). The ideal hospital scale was thus humanistic on two levels: it would give patients space and privacy within specific spaces, such as the ward, and it would be easy to navigate and familiar at an overall level. This humanistic scale operated in opposition to a compelling stereotype of the modernist or functionalist hospital, which was commonly represented as large, intimidating and unwelcoming. In 1994, an article in the *BMJ*, one of the most influential publications in the medical profession, noted that the ‘big block approach to hospitals’ of the 1960s and 1970s was increasingly being dismissed as ‘inhumane’ (Dormer 1994). As urban scholar Malcolm Miles notes, echoing this contemporary rhetoric, many twentieth-century hospitals had offered ‘new dehumanised alternatives’ to old neglected buildings and led ‘patients easily [to] feel dwarfed by impersonal vastness’ (cited in Barclay 2015).

Architects drew upon some international design trends in advocating for a turn to smaller scale hospitals. After a mid-century trip to Scandinavia, architect D. J. Petty and senior medical officer Robert Macdonald Shaw – who later played an important role in developing the Ministry of Health *Hospital Building Notes* – wrote:

There were a large number of interesting points we noticed which are only possible to touch upon. Perhaps one of the most striking was the very pleasant sense of scale achieved inside the hospitals. There was an air of quiet welcoming efficiency without any trace of the institutional feeling. We concluded that two of the reasons for this effect were the comparatively low ceiling heights … and the widespread use of naturally occurring timbers. (Shaw and Petty 1955)

Although they did not yet use the language of ‘humanistic’ design, this extract seems significant. They placed emphasis on the use of scale and ‘natural’ materials, both of which would later be advocated explicitly as features of humanistic design, to remove ‘institutional feeling’. Larger scales – echoing commercial spaces – were more commonly advocated as humanistic in the United States, where Sloane and Sloane (2003) note that the shopping mall was promoted as a model for its ‘familiarity and accessibility’. Although some UK hospitals did follow this model, medical and design literature indicates ambivalence about the ‘humanistic’ status of such design. The rise of patient as consumer was important in the UK, and linked closely with the rise of patient-centred medicine. However, models for humanistic design seemingly aligned more with Scandinavian than US trends, and drew more on the home than consumer culture.

In the UK, there were two preferred reference points for the human scale – as opposed to institutional scale – hospital: the home, or ‘domestic scale’, and the village. Such models were undoubtedly not new. Creating a domestic atmosphere, for example, had long been an explicit goal of hospitals for the aged, dying and chronically ill. However, the reframing of domestic scale as a (re)humanization tool was a new conceptualization of this design goal. In an article in *Architect's Journal*, Professor of Social Medicine Thomas McKeown (1960) directly linked hospital scale to new models of egalitarian community health in the UK, indicating also how the application of this concept related to wider social trends. Building a hospital community and focusing on healthy environments to prevent – rather than just cure – disease, he argued, required architectural as well as functional change: ‘This should be domestic rather than institutional. This change can be made by reducing the scale of buildings, by introducing variety of structure and design, and by separating hospital buildings by other amenities – shops, restaurants, amusements, etc.’ McKeown suggested building a complex of smaller buildings in line with this vision. It is noteworthy that McKeown was also well known for publications that questioned the role of medical progress in improved life expectancy, reinforcing points made above about the connection between critiques of hospitals and of general medical care.

The *BMJ* later echoed McKeown's language of the ‘hospital community’, when architect John Weeks advocated the village scale as the hospital's social and architectural model. Weeks (1985) supported the nucleus hospital as a route to the humanistic village model:

The departments that make up a hospital community are separate parts of the organisation, yet they depend on each other … [A] hospital can have the human scale and easily remembered shape of a village if the designers try, consciously, to learn from the physical characteristics of a village.

Weeks noted that the older hospitals on the pavilion plan – for all their faults – retained ‘human scale’ through their circulation patterns, but that this had been lost with new buildings designed to house ‘modern high technology medicine’. His article was therefore an explicit *re* *-*humanization agenda, and a case for new uses of scale that went beyond the scale of individual buildings. In line with McKeown's earlier plea, this idea of the human scale operating at a community level echoed changes within health care and society. Reading Weeks’ ideas in more symbolic terms, his architectural goals directly replicated the wider aims of humanistic treatment: to build a community, while acknowledging the individuality of patients within it.

The increasing emphasis on patient individuality was also evident in subtle shifts in humanistic design goals. By the very end of the twentieth century, human scale was increasingly broken down into different groups with different needs. In this framework, the human was a more individual one and situated in opposition to the perceived anonymity of large institutions. Devlin and Arneill (2003) note that medicine turned more towards created specialist building types in the final decades of the twentieth century. As part of this move, architects advocated ‘human scale’ and homely spaces to support wayfinding within units for people with dementia. Dalke, Littlefair, and Loe (2004) observe that ‘scale and perspective are crucial to understanding the design of environments for children. Some external entrances are child-centred in scale’. In line with wider shifts in the language of the human in healthcare and medical education (see Bates 2017), the communal models advocated by McKeown and Weeks gave way to uses of scale that responded more to individual patients’ needs. Overall, ideas about human scale operated in a range of ways in the late twentieth century, but always in opposition to institutional scale. It produced (complexes of) buildings and rooms that allowed for individuality and variety, hid the true size of the hospital's operations, and were not overwhelming for patients.

The human aspects of hospital design also operated in terms of aesthetics and interior design. Again, such humanistic design sought to minimize the visibility of modern medicine. Rather than counterbalancing institutional scale, such design sought to counterbalance high-technology medicine. John Weeks (1985) wrote that:

[The patient] is thankful that hospital care is available, thankful to all the men and women who administer care, and often awed by the brush with high technology medicine. But we should not assume that architectural celebration of the high technology aspects of medicine will provide a reassuring environment in most people's perception. In common terms a hospital should be “human.” But what does the word mean? Hidden in the concept there are at least two major components, one organisational and one physical.

Physically, Weeks noted, ‘a human hospital is small, architecturally familiar, nicely decorated, and made of brick with a lot of flowers and wood inside and lawns and trees outside. It has a pitched roof and ordinary sized windows’. He did not delve into his reasons for labelling these physical features as ‘human’, but implicitly aligned nature with the ‘human’ and situated both in opposition to ‘high technology’ environments. Artistic representations of nature were similarly deemed ‘humanistic’ and constructed as a counterbalance to high-technology hospital environments. The influential doctor/author William Carlos Williams, to cite a review of his work in the 1970s, promoted the value of ‘art as a humanizing agent in a technological world’ (Sherman 1976). This moment in time, in which the ‘technological world’ needed ‘humanizing’, reinvigorated a movement to incorporate both nature and arts in hospitals.

As well as representing the non-technological, arts and nature served a function for the holistic dimensions of health care by tending to the patient's spirit. Richard Burton (1990), writing on St Mary's Hospital on the Isle of Wight, equated human design in the ward with visual ‘interest’. He drew on cultural inspirations, such as Monet's ‘“waterlily” garden’ and Japanese meditation gardens, for providing patients with ‘opportunities to divert their minds from their illness’. This issue of holistic health represents a third type of humanistic design, which operated in opposition to modern biomedicine. Decades previously, under a section heading ‘humanity’, *The Lancet* (Anon. 1953) had noted that ‘since the spirit of a place is what determines the standard of the work done in it, it is well to begin by making surroundings cheerful, even if they are not ideally equipped’. In 1989, the Prince of Wales’ publication *A Vision of Britain* drew links between human-centred hospital design and the growth of holistic medicine (Glanville, Noble, and Scher 1999).

Hospital design that treated body and soul was, of course, nothing new, but again the goal of ‘humanizing’ care in this way implied a sense of loss. It was part of wider contemporary trends to advocate putting the patient as ‘whole person’ back at the centre of medical care, partly in response to modern specialization and the alleged tendency of biomedicine to reduce patients to their illnesses (or pathological body parts). The renewed emphasis on ‘cheerful’ surroundings thus emerged from a particular context, which is not directly comparable to earlier hospital design that sought – for example – spiritual or religious goals. Again, the aim of ‘humanization’ implied a counterbalance or a remedy to a problem. David Howes and Constance Classen's *Ways of Sensing* (2013) notes, in its brief section on hospitals, that ‘the hospital is a decidedly unaesthetic place … the sensory and aesthetic experiences of patients are not held to be crucial to their treatment for or recovery from illness. If nothing else, this creates an alienating divide between bodily wellbeing and sensory wellbeing’. Such a perception of the hospital as unaesthetic, and as treating only the body, has commonly underpinned those aspects of humanistic interior design that treat the soul and ‘sensory wellbeing’, such as nature and the arts.

At first the perceived relationship between nature, humanity and holistic care was to some extent grounded in assumptions about their value or based on anecdotal evidence. It gained a firmer foothold in the 1980s after Roger Ulrich's famous study showing more rapid healing for patients with a view of hospital gardens (Ulrich 1984). While there are many critiques of the limits of this study, it certainly provided an important boost for those seeking to justify attention to the human aspects of hospital design in an increasingly evidence-based world. In the same year, Hugh Baron and Lesley Greene (1984) argued strongly in the *BMJ* for the value of greater investment in hospital arts for their humanistic potential and ability to evoke emotions. In the light of these parallel trends, it is perhaps no coincidence that so many hospital arts programmes represented scenes of nature. When Southampton hospital set up a so-called ‘humanising’ committee in the early 1980s, one of its results was a colourful corridor pattern of trees (Baron and Greene 1984). Due to the general goals of such artwork, of providing distraction from the hospital environment and lifting spirits, it was rare to see challenging or abstract artworks advocated as humanizing forces in these contexts. Ideas about ‘art as a humanizing agent’ thus need to be further unpicked, as hospital design decisions indicate that not all visual art was perceived as such.

It is not possible here to do justice to the influence of the principle of ‘humanistic design’. It is for another article to explore in-depth case studies of ‘humanistic’ design and the factors that have enabled – and restricted – its implementation. It is worth briefly noting one case study of ideal practice, however. Maggie's Centres, UK drop-in centres for people with cancer that first opened in Edinburgh in 1996, physically embody all the features of ‘humanistic’ design outlined above: the buildings are non-institutional, non-biomedical and non-technological. It is perhaps no surprise that these centres are so often cited in literature on ‘therapeutic landscapes’ (for example, Butterfield and Martin 2016). Jencks himself writes that they were intended as part of a move ‘towards more humane and varied building types’ (Jencks and Heathcote 2010). The buildings emphasize light, nature and comfort. The designs are all individual, and deliberately situated in opposition to the ‘machine for healing’ model of impersonal health care. To quote Jencks further: ‘[i]nformal, like a home, a Maggie's Centre is meant to be welcoming, domestic, warm, skittish, personal, small-scaled’. Maggie's Centres also draw further attention to the materiality of ‘humanistic’ design, including surfaces that are pleasant to touch. While these are very specific and undoubtedly not representative of health care more generally, which has often only been able to implement elements of these design principles, Maggie's Centres model humanistic design in practice.

Overall, the concept of ‘humanistic’ design encompassed a wide range of different design features. These were often not new, but were increasingly understood in humanistic *terms* in the twentieth century. The most dominant or commonly cited features of humanistic design bridged the non-institutional, the non-technological and the non-biomedical. Homeliness, nature and the arts were repeated themes of literature on humanistic design because they touched upon at least two of the three categories. The precise forms that these design features would – or, rather, should – take were diverse and ranged from structural questions to interior decoration and sensory environments. What drew them together, and with the intellectual trends outlined above, was a sense of patient- (or human-) centredness. They were also defined as much by what they were *not*, as by what they were. The three trends were all constructed as remedies to the same perceived problem or loss, in broad terms at least, offering counterbalances to the apparently dehumanizing aspects of modern medicine.

## Conclusions

In 1983, the design journal *Hospital Development* observed that ‘in hospital design matters … [a] strong trend is towards “humanisation” – a principle first voiced by a few concerned people in the late 1960s. It was badly neglected then but is now in full bloom, as can be seen in both the external and internal aspects of many of the latest projects in the UK’ (Anon. 1983). The above discussion has briefly outlined some of these intellectual ‘voices’ in the 1960s and 1970s, and the forms in which these ideas entered medical literature and design practice by the 1980s. Each of these forms could be the subject of an in-depth analysis but there is also value in considering them together in relation to a bigger conceptual framework: the ‘human’. One important finding, from taking this birds-eye view, is that the idea of humanistic design was both highly influential and under-examined during the late twentieth century.

The term was used across a wide range of contexts, with particular implications in specific countries and for certain types of patient, but these differences were rarely drawn out. As in the *Hospital Development* quote above, architectural and medical commentators often referenced humanization as a ‘trend’ or ‘principle’ but failed to unpick its exact meaning and implications. Examining some of the different forms and functions of humanization indicates that the term often operated to repackage long-held ideas about holism and healthy design. This claim is not to undermine its significance. It is highly revealing that architects, designers and others were so keen to rebrand such design goals as humanistic design. This rhetoric operated – albeit in a complex way in practice – as a driver for change. The concept of the humanistic hospital tapped into a range of social, political, intellectual and economic trends; it provided an opportunity for visible resistance to (perceived) medical dehumanization.

The success or failure of these ideas is a subject for a different article, as a wide range of practical, political and economic factors limited the ability to implement humanistic design. Important questions also remain about what humanistic design meant to patients, staff and visitors, and the extent to which they were truly involved in human-centred design processes. The human in the hospital has never been a homogeneous one; some feel soothed by high technology environments, while others have specific sensory or emotional needs. It is therefore important not to assume that human-centred design has always reflected (or consulted) the diverse wishes of patients, visitors and staff. These questions are important and worthy of further scholarly attention. While the term may have waned somewhat in its social and political poignancy since the mid-twentieth century, it remains a regular feature of design and medical literature. It is crucial to pay critical attention to its meanings and forms in order better to understand some of the assumptions that underpin its currency as a design goal.

## Notes

1. Nardin (2009) traces this trend back to the early twentieth century in France, although notes that it became a newly pressing issue for government in the post-war period. The AP-HP archive and Archives Nationales in Paris also both hold extensive papers that show the ongoing interest of the government in ‘humanizing’ hospitals in the decades following this circular. A lot of this material focuses on care, but some also relates to the design of humanistic environments; as this article shows, the two issues were never truly separable.

2. Many of these debates about modernity and individualism were longstanding within international sociology of the nineteenth and twentieth centuries, in the work of Durkheim among others. As Peter Kivisto (2010) notes, ‘Riesman called attention to the threat of individualism in the middle of the past century brought about by the advent of mass society, wheras [sic] a quarter of a century later Bellah and associates saw reason to conclude that individualism had become sufficiently problematic that it was undermining community’.
